# Role of Medium-Chain Triglycerides on the Emulsifying Properties and Interfacial Adsorption Characteristics of Pork Myofibrillar Protein

**DOI:** 10.3390/foods14050796

**Published:** 2025-02-26

**Authors:** Miaomiao Shi, Muhan Zhang, Huan Bian, Daoying Wang, Weimin Xu, Suhuan Wei, Ruirui Guo

**Affiliations:** 1 Jiangsu Key Laboratory for Food Quality and Safety State Key Laboratory Cultivation Base, Ministry of Science and Technology, Nanjing 210014, China; 2022808168@stu.njau.edu.cn (M.S.); weisu@stu.njau.edu.cn (S.W.);; 2 Institute of Agricultural Products Processing, Jiangsu Academy of Agricultural Sciences, Nanjing 210014, China; 3 Key Laboratory of Cold Chain Logistics Technology for Agroproduct, Ministry of Agriculture and Rural Affairs, Nanjing 210014, China

**Keywords:** emulsion properties, myofibrillar protein, medium-chain triglycerides, interfacial protein adsorption

## Abstract

Medium-chain triglycerides (MCTs) have been known to have multiple health benefits in treating metabolic disorders and reducing the incidence of obesity. In the present study, the partial replacement of lard with MCTs assisted by ultrasound treatment on the emulsifying stability and adsorption behavior of myofibrillar protein (MP) was investigated. The results revealed that ultrasound-assisted MCT emulsion had better emulsifying activity and emulsion stability than other groups. MCTs with ultrasound treatment considerably lowered the particle size, facilitated the formation of much smaller and more homogeneous emulsion droplets, and enhanced the oxidative stability of the emulsion. The emulsion had a pseudo-plastic behavior determined through static and dynamic rheological studies, and the MCT emulsion exhibited a larger viscosity and a greater storage modulus (G′) compared with the lard emulsion. MCTs could promote protein adsorption levels at the O/W interface, forming a dense interfacial protein film. The surface hydrophobicity and reactive sulfhydryl content increased, accompanied by the transformation of α-helix and β-turn structure to β-sheet and random coil structure, indicating MCTs combined with ultrasound-induced unfolding and crosslinking of MP at the interface. The results suggested that MCTs may have the potential to enhance emulsifying properties in emulsion-type meat products.

## 1. Introduction

An oil-in-water (O/W) emulsion is a heterogeneous system in which the discrete oil phase is dispersed in a continuous aqueous phase [1]. Myofibrillar protein (MP) is a prominent functional protein in emulsion-type meat products that can stabilize oil droplets and adsorb at the O/W interface. With an amphiphilic nature, MP molecules can interact with each other or with oil by hydrophobic interaction to strengthen the mechanical properties of interfacial films and prevent the flocculation of the droplets [2]. Animal fat is often incorporated into the MP matrix during the processing of meat products, which not only improves the textural properties and flavor of meat products but also plays important roles in the emulsification, water-holding capacity, and gel-forming ability of meat emulsions [3]. However, excessive animal fat intake is known to increase the risk of some chronic disorders such as obesity, hypertension, or cardiovascular diseases [4]. Replacing animal fat in various meat products with cereal flours, dietary fibers, proteins, and vegetable oils has gained great interest in recent years to improve the storage performance and health attributes of meat products [4].

The formation of interfacial films and emulsion stability depend on the physicochemical properties of protein and oil, and the differences in oil properties, including hydrogen chain length, saturation, viscosity, and polarity, can influence the dynamic adsorption, arrangement, and deformation of protein particles at interfaces and the emulsion stability [5]. Zhang et al. [6] found the stability of an emulsion is enhanced when the oil polarity increases within a range because of the lower interfacial tension of the polar lipids, whereas a further increase in polarity leads to a collapse of the interfacial film. In a study by Han et al. [7], fatty acids with low degrees of unsaturation can facilitate the adsorption of proteins at the interface and contribute to the compact gel-like network structures, ultimately enhancing the emulsification stability. However, Zheng et al. [8] reported that fat containing more neutral fatty acids, polyunsaturated fatty acids (PUFA), and long-chain fatty acids is more conducive to the steady of the emulsification system, while the more short-chain fatty acids and saturated fatty acids are not beneficial for the stability of the emulsion.

Medium-chain triglycerides (MCTs), containing fatty acid chains in the range of 6–10 carbon atoms, are more easily digested and quickly metabolized compared with long-chain triglycerides (LCTs) [9]. Owing to their direct passage through the portal vein to the liver, they are less likely to accumulate in fat tissue [9]. The MCT-containing ice creams, cooking oil, yogurt, etc., have been commercially available in the market to reduce the calorific value of food [10]. The lower molecular weight, polarity for both water and lipids, and lower viscosity make them useful in the food industry as a solvent to dissolve food color, flavor, and bioactive molecules, and they are used for cooking purposes because of their low smoke point [9,10]. The MCT-containing emulsion has been developed to increase freeze–thaw stability, preparation of bio-compatible emulsions, and provide nutritional value [11,12]. However, the utilization of MCTs in meat products and the functional properties of MCTs in meat emulsification systems are rarely explored.

Many emulsification technologies have been used to produce stable homogeneous emulsions, including mechanical agitation, high-pressure homogenization, microfluidization, etc. [13]. Ultrasound has been widely applied as an effective emulsification technology in the development of emulsions because of the advantages of being environmentally friendly and highly efficient. Ultrasound has a cavitation effect and generates shear force, mechanical force, and other physical effects to decrease emulsion droplet size, enhance emulsion stability, and alter the functional properties of proteins [14,15]. The use of ultrasound to treat proteins can effectively improve the interfacial properties of proteins by exposing hydrophobic groups, increasing surface charge, and altering the molecular structure [16,17]. The application of ultrasound on a pre-emulsion favored the interaction between oil droplets and the stabilization of pickling emulsion against coalescence [14]. Therefore, the aim of the current study was to investigate the partial replacement of lard with MCTs in combination with ultrasound on the interfacial adsorption behavior and emulsifying properties and further explore the interface-induced conformation changes in protein and the stabilization mechanism. This study will provide a reference for healthy emulsified meat products and more rational control of emulsion stability.

## 2. Materials and Methods

### 2.1. Materials

The fresh lean pork (12 h postmortem, pH 5.51, moisture 72.90%, fat content 2.60%, protein content 22.63%) used in this research was obtained from a local commercial supermarket (Nanjing, China), lard and MCTs (>98% purity) were obtained from Yuanye Bio-Technology Co., Ltd. (Shanghai, China). All additional reagents utilized in the experiment were of analytical grade. The visible connective tissues and fat were removed from the pig foreleg muscle, the meat was then packaged and kept at 4 °C for extraction of MP within the same day.

### 2.2. Extraction of Myofibrillar (MP) Proteins

MP was extracted from pig foreleg meat according to the method of Park and Xiong [18] with slight modifications. Briefly, the visible fat and connective tissue were removed, and the meat was minced with a grinder. The minced meat was mixed with phosphate buffer (10 mM, 0.1 mol/L NaCl, 2 mmol/L MgCl_2_, and 1 mmol/L EGTA, pH 7.0) in a 1:4 (*w*/*w*) ratio using a homogenizer with a S25N 18 mm diameter cylindrical blade (IKA, Ultra Turrax T25, Staufan, Germany) at 10,000 rpm for 2 min. The homogenized solution was then centrifuged at 2000× *g* at 4 °C for 15 min. Then, the precipitate was washed with 4 volumes of the same buffer two more times, and centrifugation was conducted in the same condition indicated above. Finally, the myofibril suspension was filtered through 4 layers of gauze. The protein concentration was measured using the biuret method with bovine serum albumin (BSA) as the standard using a protein assay kit (Jiancheng, Nanjing, China).

### 2.3. Preparation of MP Emulsion

Emulsions with lard or MCTs were prepared at an oil volume fraction (φ) of 20%. Before preparation of the emulsions, lard was liquefied in a water bath at 35 °C. 20 mL lard or lard (10 mL) and MCTs (10 mL) were mixed with 80 mL 15 mg/mL MP solution, then the mixture was placed in a 30 °C water bath for 5 min and homogenized at 10,000 rpm for 2 min with a S25N 18 mm diameter cylindrical blade (IKA, Ultra Turrax T25, Staufan, Germany). The emulsions prepared with lard alone and by replacing 50% of lard with MCTs (10 mL lard and 10 mL MCTs) were marked as L and LM, respectively. Then, a portion of the L and LM samples were subjected to ultrasound treatment (SCIENTZ-IID, Ningbo Xinzhi Ultrasonic Technology Co., Ltd., Zhejiang, China) with a 12 mm diameter probe immersed in the emulsion (output power of 180 W, 20 kHz) for 5 min (on-time 2 s and off-time 3 s). The L and LM emulsions after ultrasound treatment were labeled as LU and LMU, respectively.

### 2.4. Emulsifying Activity Index (EAI) and Emulsion Stability Index (ESI)

The EAI and ESI were determined according to Li et al. [17]. Aliquots of prepared emulsions (0.05 mL) were dispersed into 5 mL of 1 mg/mL SDS (sodium dodecyl sulfate), and the absorbance was measured at 500 nm using a spectrophotometer (Biotek, Winooski, VT, USA), with SDS solution as blank. The EAI was calculated by the equation as follows:

$$\mathrm{EAI}\left(\frac{m^{2}}{g}\right)=\frac{2.303\times 2\times A_{0}\times N}{c\times \Phi \times 10^{4}}$$

where A_0_ represents the absorbance of the emulsion measured right at 0 min after its formation, N is the dilution factor, c denotes the protein concentration (g/mL) in the solution, and Φ accounts for the oil’s volume fraction. ESI was calculated by the equation as follows:

$$\mathrm{ESI}(\%)=\frac{A_{0}}{A_{10}}\times 100$$

where A_0_ and A_10_ are the absorbance at 500 nm immediately and after 10 min, respectively.

### 2.5. Particle Size and ζ-Potential

The droplet size and ζ-potential of the emulsion were measured with a nanoparticle size potentiometer (Nicomp Z3000, Particle Sizing System, Port Richey, FL, USA). The emulsion was added to a cuvette immediately after preparation. The refractive indexes were set at 1.330 and 1.475 for the continuous and dispersed phases, respectively.

### 2.6. Oxidative Stability

The degree of lipid oxidation was analyzed by 2-thiobarbituric acid-reactive substances (TBARS), the secondary oxidation product. The emulsion was incubated at 37 °C for 0 days and 3 days, respectively. Emulsion (2.0 mL) was mixed with 4.0 mL of TBA reagent and heated in a 95 °C water bath for 15 min, then cooled to room temperature. A volume of 3 mL of the reaction mixture was mixed with 2 mL of chloroform (chloroform glacial acetic acid, 2:3, V/V) and centrifuged at 12,000 rpm for 15 min. The absorbance of supernatants was measured at 532 nm. The malondialdehyde was calculated from a standard curve prepared by acidification of 1,1,3,3-tetraethoxypropane (TEP) from 0.02 μg/mL to 0.3 μg/mL.

### 2.7. Rheological Properties of Emulsion

The rheological properties of samples were measured by the MCR302 rheometer (Anton Paar, Grraz, Austria). A parallel plate with a diameter of 25 mm and a gap of 1 mm was used. By setting the shear rate between 1 and 100 s^−1^, viscosity was measured to confirm the emulsion’s shear thinning behavior. The storage modulus (G′) and loss modulus (G′′) were determined using a dynamic frequency sweep within the frequency range of 1 to 100 rad/s at a strain level of 2%. The measurement was carried out at 25 °C, and the emulsion’s rheological properties were measured on the 0 and 3 days.

### 2.8. Emulsion Microstructure

A droplet of each sample was placed on a microscope slide and gently covered with a glass slide. An optical microscope (Nikon E200, Tokyo, Japan) was used to observe the micromorphology of droplets in the emulsion under a 40× objective lens.

### 2.9. Chemical Forces

The chemical forces were measured by adding 5 mL of the following solutions to 1 mL samples: 0.05 mol/L NaCl (SA), 0.6 mol/L NaCl (SB), 1.5 mol/L urea with 0.6 mol/L NaCl (SC), 8.0 mol/L urea with 0.6 mol/L NaCl (SD), and 8.0 mol/L urea with 0.6 mol/L NaCl and 1.5 mol/L β-mercaptoethanol (SE). The homogenates were mixed well, left to stand for 1 h, and centrifuged at 12,000× *g* for 15 min. The protein concentration was determined using the biuret method. The ionic bonds were quantified from the differences in the solubility between SB and SA samples, the hydrogen bonds were calculated from the differences in the protein concentrations between the SC and SB samples, the hydrophobic interactions were quantified from the differences in the protein concentrations between SD and SC samples, and disulfide bonds were determined by the differences between the solubilized protein in the SE and SD samples.

### 2.10. Secondary Structure of MP

MP suspensions (0.15 mg/mL) were diluted with a phosphate buffer (10 mmol/L, 0.6 mol/L NaCl, pH 7.0) and placed in a 0.1 cm quartz cuvette for analysis using circular dichroism (CD) on a Jasco J-715 device (Jasco Corp., Tokyo, Japan) operated with a 0.25 s response time, scanning range from 190 to 260 nm at the speed of 100 nm/min. The secondary structures of MP were calculated as proportions of α-helix, β-sheet, β-turn, and random coil structures.

### 2.11. Reactive Sulfhydryl (SH) Group of MP

The reactive sulfhydryl content of MP was measured as described previously by Ellman [19] with modifications. MP samples were diluted to 2.0 mg/mL with phosphate buffer (10 mmol/L, 0.6 mol/L NaCl, pH 7.0), and 2.0 mL MP suspensions were thoroughly mixed with 5,5′-dithiobis-2-nitrobenzoic acid (5 μL). Following a 20 min incubation at 25 °C, the absorbance of the MP sample was measured at a wavelength of 412 nm. The reactive SH group content was determined using an extinction coefficient of 13,600 M^−1^cm^−1^.

### 2.12. Surface Hydrophobicity

The compound 8-anilino-1-naphthalene sulphonic acid (ANS) was used as a hydrophobic probe to evaluate the hydrophobicity of the MP samples. The samples were adjusted with 10 mmol/L phosphate buffer to achieve concentrations ranging from 0.2 mg/mL to 1.0 mg/mL. Afterward, 4.0 mL of each sample was combined with 100 μL of an 8.0 mmol/L ANS solution and left to incubate in the dark for 20 min. The fluorescence intensity was recorded with an excitation wavelength of 390 nm and an emission wavelength of 470 nm, with excitation and emission slits set at 10 nm. The hydrophobicity of MP samples was measured by the initial slope of the linear regression equation when fluorescence intensity was plotted against protein concentration.

### 2.13. Determination of Interfacial Proteins and Sodium Dodecyl Sulfate-Polyacrylamide Gel Electrophoresis (SDS-PAGE) Analysis

The evaluation of adsorbed protein and non-adsorbed protein was performed as described by Li et al. [17] with slight modifications. The emulsion was centrifuged at 15,000× *g* for 30 min at 4 °C, and then the supernatant that contained the non-adsorbed protein was transferred to another centrifuge tube. The non-adsorbed protein concentration was determined with the biuret method, and the adsorbed protein content was determined by subtraction of the non-adsorbed protein content from the emulsion. The emulsified layer with adsorbed proteins on the top was resuspended in 0.6 M NaCl buffer and centrifuged at 15,000× *g* for 30 min under 4 °C. The above steps were repeated three times. Finally, the obtained non-adsorbed and adsorbed protein was subjected to SDS-PAGE.

Samples were analyzed in a Mini-PROTEAN Electrophoresis system (Bio-Rad Co. Ltd., Hercules, CA, USA), using 10% and 4% for separating and stacking gels, respectively. The electrophoresis was run at a voltage of 120 V. After completion, the gel was stained with Coomassie Brilliant Blue solution (0.25% Coomassie Brilliant Blue R-250, 50% methanol, and 10% glacial acetic acid) for 2 h, and then decolorized with a decolorizing solution (containing 22.5% absolute ethanol, 2.5% glacial acetic acid) until the background is clear. Images of the gel were captured by a Gel Imager (Tanon Co., Shanghai, China).

### 2.14. Statistical Analysis

All experiments were carried out in triplicate. Experimental data were represented as mean ± standard deviation and analyzed using the SPSS statistical software package 26.0 (IBM, Chicago, IL, USA). One-way analysis of variance (ANOVA) was used to analyze these data, with a *p* < 0.05 considered statistically significant.

## 3. Results and Discussion

### 3.1. Characteristics of the Emulsions

**Figure 1:**
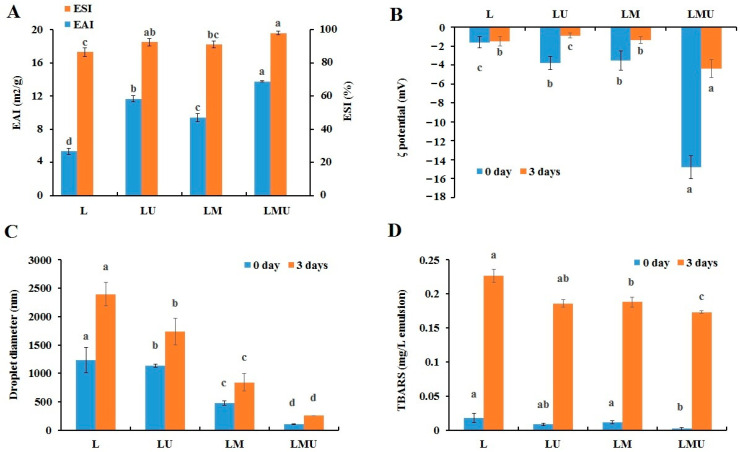
Emulsifying activity index (EAI) and emulsifying stability index (ESI) (**A**), ζ potential (**B**), droplet diameter (**C**), and TBARS (**D**) of the emulsions prepared with MP and lard or MCTs with or without ultrasound treatment. Significant differences between emulsions within storage days are denoted by different letters (*p* < 0.05).

The changes in EAI and ESI of MP under different treatments are shown in Figure 1A. EAI reflects the adsorption capacity of protein at the oil-water interface, while ESI is the ability of emulsion to maintain dispersion and stability [3]. The EAI of LM (9.41 m^2^/g) was considerably higher than that of L (5.33 m^2^/g), and LMU (13.78 m^2^/g) was significantly higher than that of LU (11.68 m^2^/g) (*p* < 0.05). Similar to EAI, LMU exhibited the highest ESI, followed by LU, LM, and L. The results indicated that both partial replacement of lard with MCTs and ultrasound could effectively enhance the emulsifying activity and stability of MP.

The ζ-potential reflects the surface charge density and interactions between proteins and fat [20]. The ζ-potential of emulsion under different treatments is shown in Figure 1B. On day 0, the absolute ζ-potential value increased significantly when lard was partially replaced by MCTs (*p* < 0.05) and further increased when ultrasound was applied (*p* < 0.05), indicating that the emulsion became more stable. On day 3, the absolute ζ-potential in all groups declined; there was no remarkable difference between the L and LM groups (*p* > 0.05), but ζ-potential in the LMU group was significantly higher than that in the LU group (*p* < 0.05).

After a 3-day storage period, the droplet size of all emulsions was increased compared with freshly prepared emulsions, reflecting the aggregation of droplets during storage (Figure 1C). The droplet size significantly decreased from 1230.47 nm in the L group to 481.25 nm in the LM group, and the ultrasound treatment promoted the smaller emulsion droplet formation to 104.58 nm at day 0. On day 3, the droplet size manifested a similar trend. The MCTs are comprised of saturated fatty acids, while lard is abundant in unsaturated fatty acids, and the molecular weight of MCTs is smaller than that of LCTs. The results were in accordance with the previous study that the saturation degree of fatty acids has important effects on the droplet size of emulsions [7]. With the increase in unsaturation degree, the emulsion size rises along with a decline in emulsion stability [7]. The results were also attributed to the higher polarity of MCTs. It has been reported that emulsions made with oils of higher polarity have smaller sizes and better emulsion stability since more polar oil is more potent in reducing interfacial tension and promoting droplet fragmentation [6].

Lipid oxidation can have negative effects on emulsion stability and the quality of emulsified meat products. TBARS represents the number of secondary oxidation products formed during lipid oxidation. In the present study, the oxidation degree increased rapidly in all groups after 3 days of storage, the incorporation of MCTs significantly reduced the TBARS value in emulsions compared with that prepared by lard after 3 days of storage (Figure 1D), and the oxidation level of the emulsion was dramatically decreased after ultrasonic treatment (*p* < 0.05). A reason for the higher oxidative stability of LMU was that MCTs contained more saturated fatty acids, which could resist the oxidative attack; another reason was proteins stabilized on the interface were beneficial to form a physical interface film and tightly encapsulated the oil droplets, which limited the exposure of oil and diffusion of free radicals or pro-oxidants [21]. In a study by Liu et al. [12], MCTs can enhance the freeze–thaw stability of the emulsion. MCTs have also been used as the biocompatible oil phase in the development of nutrition formulation, nutraceuticals, and encapsulation of active ingredients [11]. MCTs have many advantages in emulsions to improve long-term storage stability and have broad practical applications in the food industry.

### 3.2. Rheological Properties of Emulsion

**Figure 2:**
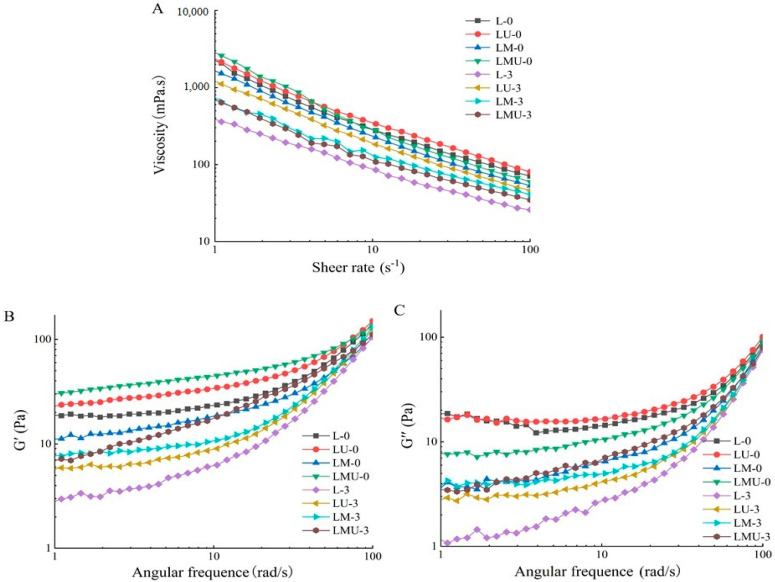
Rheological properties of emulsions stabilized by lard or MCTs freshly prepared and after 3 days of storage at 37 °C. (**A**) apparent viscosity versus shear rate; (**B**) storage modulus G’ with angular frequency from 1 to 100 rad/s; (**C**) loss modulus G″ with angular frequency from 1 to 100 rad/s.

As presented in Figure 2A, the viscosity of all groups decreased with the increment of shear rate and displayed a shear thinning phenomenon, indicating that all emulsions were non-Newtonian pseudoplastic fluids. At the low shear rate before the storage, the viscosity of LMU was higher than those of other groups, but with the increase in shear rate, LU had the highest viscosity. After storage for 3 days, the viscosity of all groups decreased as compared with that of on day 0. The smaller oil droplets usually have a greater specific surface area that can enhance intermolecular interactions and increase the viscosity [22]. A previous study revealed that viscosity is related to the droplet size and oil type, and MCT oil with smaller particle size have the highest viscosity [22]. Kim et al. [23] evaluated the meat emulsion prepared with different oil types and observed that a higher apparent viscosity was correlated with stronger emulsion stability. The cavitation effects caused by ultrasound treatment generate bubbles that lead to droplets breaking into small droplets and, thereby, change the attractive forces or repulsive forces between droplets, which increases the viscosity of the emulsions [14].

The behavior of the storage modulus (G’) and the loss modulus (G’’) of the emulsion during frequency sweeps are presented in Figure 2B,C. The value of G’ of all emulsions was larger than G’’, demonstrating that the emulsion mainly displayed elastic gel performance and a network was generated within the emulsion [16,24]. G’ of each emulsion decreased after 3 days of storage in comparison to that of the emulsions on day 0, indicating the weakened network structure during storage. The aggregation and coalescence of emulsion droplets during storage increases droplet size and decreases the interfacial area; therefore, the inter-droplet interaction is decreased, and the gel network structure is weakened [24]. The introduction of MCTs and ultrasound treatment increased G’ of emulsions both on day 0 and day 3. It has been suggested that gel strength is primarily governed by the particle network at droplet interfaces, and in the continuous phase, the higher G’ is attributed to the enhanced droplet interactions and higher network rigidity [25]. Boutin et al. [26] found that the size of the emulsion droplets could influence the rheological behavior of the system; the smaller the emulsion droplets, the more uniform and regular the gel matrix network. The results were also in line with the results above.

### 3.3. Microstructure and Macroscopic View of Emulsions

**Figure 3:**
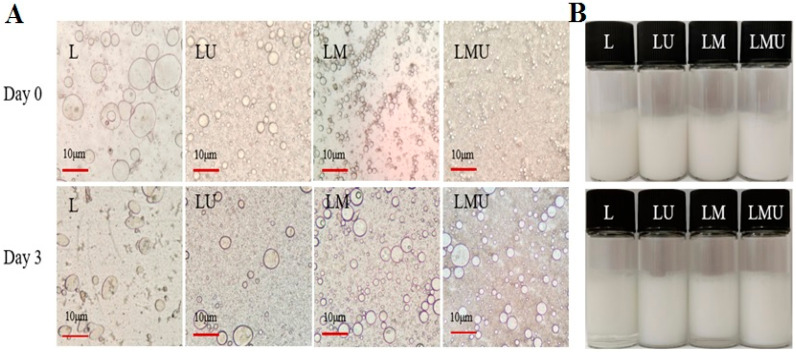
Microstructure (**A**) and appearance (**B**) of emulsions under different treatments after storage for 0 and 3 days at 37 °C.

Figure 3A illustrates the microscopic images of emulsions freshly prepared and after storage for 3 days. The emulsion in the L group had a relatively large particle diameter and uneven droplet distribution; emulsions in the LM group produced obviously clustered smaller droplets forming a chain-like structure, and in the LMU group, the size of particles further decreased and was more evenly dispersed. The results were consistent with the droplet size results. Creaming is one of the most common instability phenomena in emulsions because the oil phase is less dense than water and rises and accumulates at the top. Fresh emulsions in each group showed no significant creaming, and after storage for 3 days, the LU and LMU groups maintained no creaming, but stratification was observed in the L and LM groups (Figure 3B). MCTs have higher miscibility with water, but lard has a low affinity with water, which can induce the coalescence of droplets and the formation of large droplets [3]. Similar findings were reported by Chen et al. [27] that the addition of MCTs improves the stability of emulsions. They hypothesized that the adsorbed protein layer is cross-linked, and this increases the emulsion stability against coalescence and Ostwald ripening by forming a rigid interfacial coating. The microstructure of L and LM showed partial aggregation and droplet coalescence, while in LU and LMU groups, the more homogeneous emulsion was formed with better stability. The impact of ultrasound on decreasing particle size and enhancing emulsifying properties has been reported, and this effect is due to the cavitation and shear force along with turbulence by ultrasound in the disruption and dissociation of myofibrillar proteins [17].

### 3.4. Characteristics of Interfacial Proteins

**Figure 4:**
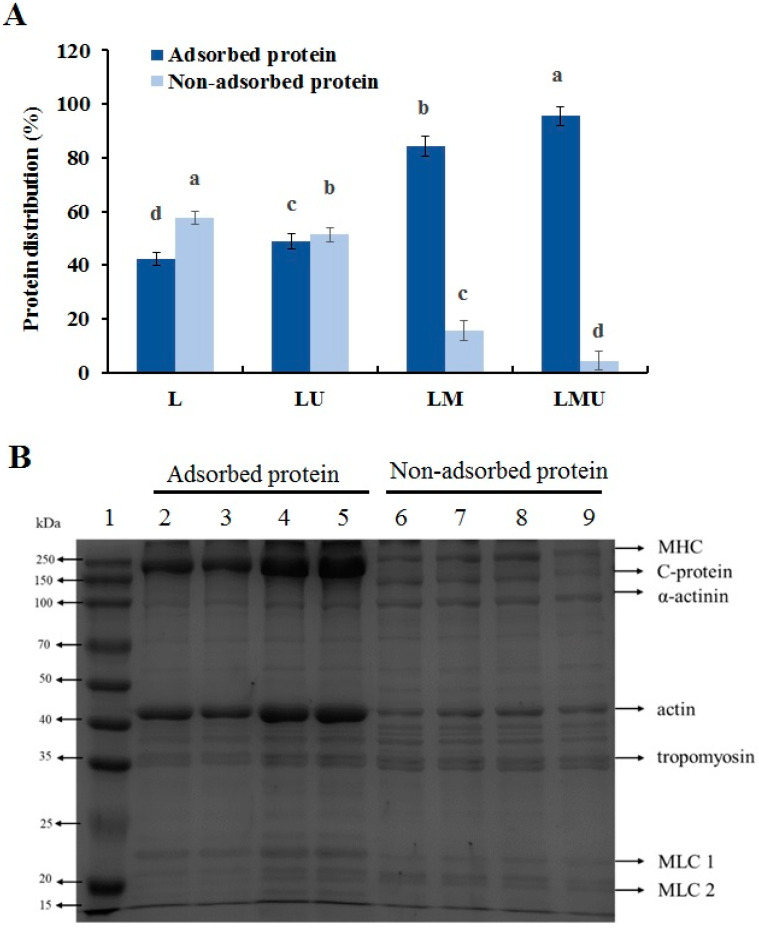
Adsorbed protein and non-adsorbed protein of emulsions after different treatments. (**A**) percentages of non-adsorbed protein (in the aqueous layer) and adsorbed protein (in the emulsion layer), different letters in the same protein groups indicate significant differences (*p* < 0.05). (**B**) SDS-PAGE of non-adsorbed protein and adsorbed protein of emulsion. Lane 1: standard marker (kDa); Lane 2–5: adsorbed protein in the L, LU, LM, LMU groups; Lane 6–9: non-adsorbed protein in the L, LU, LM, LMU groups. MHC: myosin heavy chain; MLC: myosin light chain.

To characterize the proteins adsorbed at the O/W interface, the amount of adsorbed protein and non-adsorbed protein was determined, and the protein profiles were examined by SDS-PAGE. In the L group, the non-adsorbed protein level was higher than the adsorbed protein (Figure 4A), the incorporation of MCTs significantly increased the adsorbed protein content to 84.43% (*p* < 0.05), and LMU had the highest content of adsorbed protein, which accounted for 95.56% of total protein. An increase in the level of adsorbed proteins leads to a stronger interface layer, allowing proteins to interact with oil or adjacent interfacial proteins through hydrophobic interactions, thereby enhancing the mechanical strength of interfacial films and stabilizing emulsion droplets against coalescence [28]. The decrease in particle size can promote structural flexibility and mobilization, rapidly form the interfacial protein layer, and decrease the interfacial tension, increasing the adsorbed protein proportion and stability of the emulsion [6,17].

As shown in Figure 4B, the myosin heavy chain (MHC) and actin were the major components in both adsorbed and non-adsorbed proteins, with additional proteins such as myosin light chain 1 (MLC 1), C-protein, MLC 2, α-actinin, and tropomyosin also displayed in the bands. The number of SDS bands for each group of emulsions did not change, indicating any alteration in protein composition, but there were differences in protein content. Corresponding with the protein content, the intensity of MHC and actin band increased from adsorbed protein in MCTs and ultrasound stabilized emulsion compared with lard and that without ultrasound treatment, while tropomyosin, C-protein, and α-reactive protein primarily exist in the form of non-adsorbed proteins. The results were in agreement with previous studies that both non-adsorbed proteins and adsorbed proteins generally comprised of MHC and actin, and MHC and actin content increased in adsorbed protein after ultrasound treatment, while tropomyosin remained in the aqueous layer [17,29]. The results suggested that myosin and actin may constitute the interfacial protein membrane and play important roles in the stabilization of emulsions. Interfacial protein membranes can emulsify and fix fat particles, and the gel structure can bind and limit the flow of fat and water; the elastic gel and increased interfacial protein membrane formation by MCTs and ultrasound can improve the water-holding and oil-holding capacity and also the texture of the emulsified meat products [7].

### 3.5. Physicochemical Properties of MP

**Figure 5:**
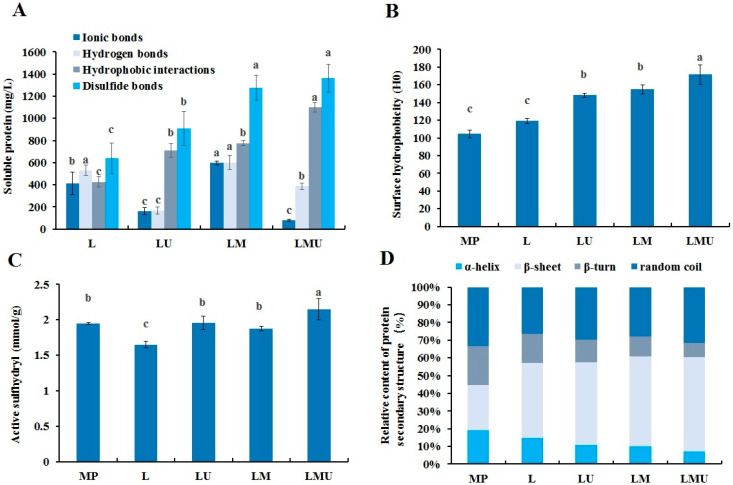
Molecular interactions (**A**), surface hydrophobicity (**B**), active sulfhydryl (**C**), and secondary structure (**D**) of MP in different emulsions. Significant differences between emulsions are denoted by different letters (*p* < 0.05).

Figure 5A shows the MP interactions in emulsions under different treatments. The hydrophobic interactions and disulfide bonds increased when MCTs were added and ultrasound was applied. The ionic bonds and hydrogen bonds decreased under ultrasound treatment, which was probably derived from the cavitation effect and shear stress of ultrasound that disrupted the non-covalent interactions within proteins and induced the rearrangement of protein structures [29]. The molecular dynamic simulations predicted by Bergfreund et al. [30] showed that upon adsorbed to interfaces, the proteins orient their hydrophobic cavity towards the oil phase, undergoing a rapid unfolding that leads to the reorganization of hydrophobic residues towards the oil accompanied by hydrophilic residues towards the aqueous phase. The filamentous MP and globular protein adsorbed to the interfaces associate with neighboring interfacial proteins via hydrophobic interactions and disulfide bonds to form a viscoelastic film [31]. The increase in hydrophobic interactions contributes to the MP gel matrix properties by accelerating the adsorption of MP to the O/W interface, while disulfide bonds promote the strong crosslinking of MP and enhance the viscoelasticity of the interfacial protein film [32]. Bergfreund et al. [30] also found a correlation between oil polarity and the surface pressure of proteins; the increased interactions of the oil with the protein can lower the interfacial pressure and energetic force of unfolding.

The surface hydrophobicity of proteins could determine the protein adsorption at the O/W interface [30,33]. As depicted in Figure 5B, the incorporation of MCTs in emulsion assisted by ultrasound treatment significantly increased the surface hydrophobicity of proteins, suggesting that the MCTs interacted with proteins and induced the unfolding of the structure and exposure of hydrophobic groups of proteins [7]. The higher surface hydrophobicity may contribute to the crosslink between neighboring proteins and protein–lipid to form the dense interfacial film [33]. The presence of SH groups at the interface can facilitate the formation of a covalently bonded, intricately interconnected, and viscoelastic network, effectively hindering droplet coalescence [28]. As shown in Figure 5C, LMU had a significantly higher content of active SH groups compared with other groups, indicating more unfolding and exposure of more interior SH groups to the surface. The exposure of more SH groups at the O/W interface contributed to the increased formation of disulfide linkages among proteins and enhanced the emulsion elasticity [34].

The secondary structure of MP was assessed by CD spectra and the content of each structure was calculated in Figure 5D. The content of α-helix and β-turn reduced in the LM group compared with that of the L group, and contents of β-sheet and random coils increased, indicating MCTs resulted in the destruction of the ordered structure of α-helix and the alteration to β-sheet and random coils. The ultrasound treatment further declined the α-helix and β-turn contents and increased β-sheet and random coils contents in the LMU group compared with the LM group, as also reported in another study [17]. The α-helix structure is predominantly stabilized through intra-molecular hydrogen bonds; thus, the decreased α-helix structure implies the rupture of the hydrogen bonds and interface-induced denaturation [35]. The untwisting and unwinding of the α-helix structure can cause the exposure of more groups to the exterior and trigger the protein cross-linking [36]. It was pointed out that more flexible proteins are prone to rearrange more quickly at O/W interfaces, efficiently reduce the interfacial tension, and improve the emulsifying properties [37]. The results demonstrated that MP might undergo reconstruction and crosslinking through hydrophobic interaction and disulfide bonds when penetrating the MCTs and adsorbed MP was more unfolded with the unwinding of a part of the helical tail to form multilayer interfacial films.

## 4. Conclusions

This study demonstrated that MCTs combined with ultrasound significantly improved the physical and oxidative stability of emulsions by forming a thicker interfacial layer which increased the interfacial protein adsorption than the lard emulsion. The unfolding of MP, exposure of hydrophobic residues and sulfhydryl groups, and transformation of α-helix and β-turn to β-sheet and random coils promoted the protein-protein and protein-oil interaction at the interface to develop a viscoelastic film. The structural alteration of MP and increased level of interfacial proteins contributed to the improvement in the emulsifying properties and emulsion stability. MCTs can be employed as a preferable replacement for lard in the production of emulsified meat products with improved quality and health benefits.

## Data Availability

The original contributions presented in the study are included in the article, further inquiries can be directed to the corresponding authors.
